# The Prevention of Typhoid Fever in Armies

**Published:** 1902-11-08

**Authors:** 


### THE PREVENTION OF TYPHOID FEVER IN ARMIES.

The long-expected discussion upon the proposals made by Dr. Leigh Canney for the prevention of typhoid in armies by providing the troops with boiled water took place at the last meeting of the Medical Society. Dr. Canney set forth his now well-known views, holding that it was highly improbable that dust or flies could give rise to an epidemic of typhoid fever in a camp with properly organised latrines, that the theory of convection could not be maintained, and that the main avenue of introduction of the bacillus into the human body was the water supply. He further said that the spread of the disease by the subsidiary channels—flies, dust, and contact—only became factors of any importance under conditions of the grossest neglect of sanitation. In the discussion which followed, however, much doubt was thrown upon Dr. Leigh Canney's main conclusions, at any rate so far as concerned the exclusive influence of water in disseminating the disease. Everyone, of course, admitted the effectiveness of polluted water as a means of distributing typhoid infection, but most of the speakers differed from him in regard to the exclusive or dominant influence of water supply in the circumstances of warfare, to which Dr. Canney's remarks had been specially directed.

Dr. C. Childs said that it was admitted on all sides that typhoid infection might be conveyed through various channels, but he took exception to Dr. Canney's statement that if water avenues were closed all the others might be neglected. Many outbreaks were undoubtedly due to water convection, but endemic and sporadic cases could be better explained in other ways. Direct infection was, he thought, more common than was generally suspected. He criticised the data drawn from Egypt and South Africa upon which Dr. Canney had based his conclusions, and held that while water was undoubtedly one, perhaps the chief, means of conveying the infection, dust, flies, and general insanitary conditions were also potent factors.

Dr. Edward Squire pointed out that in the campaign in Suakin the entire water taken by the troops was distilled, and thus thoroughly disinfected, yet the incidence of enteric fever was very considerable.

Major R. H. Firth, R.A.M.C., drew attention to the very different conditions which existed in the Army, especially when in the field, compared with those of civil life, holding that Dr. Canney took too little account of dust, flies, and other means of conveying the disease. It was in tent life, when the men were very closely packed together, that the greatest danger arose. He also insisted upon a very important point, namely, the necessity of correct and trustworthy methods of disposing of excreta being practised by the troops in time of peace, for, as things are, he said, in peace time the disposal of the excreta was performed by contractors, and consequently as soon as the soldiers took the field they were at a loss to know what to do with them.

As emphasising the importance of a pure water supply, which we suppose no one doubts, the remarks of Surgeon-General Sir A. C. de Renzy were interesting, in that he drew attention again to the enormous reduction in the death-rate from typhoid fever which had taken place at Fort William, Calcutta, at Peshawur, and other places on the introduction of a pure water supply. It is to be noted, however, that great as was the effect of the change in the water supply at these places—proving that the bad water had been entirely responsible for a large portion of the disease which had existed—still the fact that there was a considerable residuum of cases upon which the new water supply had no effect whatever—an irreducible minimum soon being reached beyond which no improvement took place—was an equally definite proof that the water was not responsible for the whole of the mischief, and that a considerable proportion of the cases occurred quite independently of water-borne infection. The general opinion seems to be—the common-sense one—that to obtain protection from typhoid fever very greatly improved methods of general sanitation must be insisted upon in military camps, and that among other means to health a supply of pure water must be provided. Indeed, this may be taken as the final outcome of the discussion, for Dr. Canney himself has now added to his demand for a "water section" one for a "pioneer section" to superintend the management of soil, the establishment of latrines, and so on, and holds that "if the water supply were in the first place protected, *and the excreta disinfected*, then the risk of epidemics in sanitary camps might be disregarded." Which goes without saying.

